# Safety and efficacy of amphotericin-B deoxycholate inhalation in critically ill patients with respiratory *Candida* spp. colonization: a retrospective analysis

**DOI:** 10.1186/s12879-014-0575-3

**Published:** 2014-10-28

**Authors:** Patrick J van der Geest, Erik I Dieters, Bart Rijnders, Johan AB Groeneveld

**Affiliations:** Department of Intensive Care Medicine, Erasmus Medical Centre, Gravendijkwal 230, Rotterdam, 3015 CE The Netherlands; Department of Medical Microbiology, Erasmus Medical Centre, Gravendijkwal 230, Rotterdam, 3015 CE The Netherlands

**Keywords:** Amphotericin B, Lung injury, Pneumonia, Efficacy, Safety, Mechanical ventilation

## Abstract

**Background:**

*Candida* spp. are frequently cultured from the respiratory tract in critically ill patients. Most intensivists start amphotericin-B deoxycholate (ABDC) inhalation therapy to eradicate *Candida* spp. from the respiratory tract. However, the safety and efficacy of this treatment are not well established. The purpose of this study was to assess the safety and efficacy of ABDC inhalation for the treatment of respiratory *Candida* spp. colonization in critically ill patients.

**Methods:**

All non-neutropenic patients admitted into the intensive care unit (ICU) of a university hospital from December 2010–2011, who had positive *Candida* spp. cultures of the respiratory tract for more than 1 day and required mechanical ventilation >48 h were retrospectively included. The decision to start ABDC inhalation had been made by attending intensivists on clinical grounds in the context of selective decontamination of the digestive tract. Infection characteristics and patient courses were assessed.

**Results:**

Hundred and thirteen consecutive patients were studied. Fifty-one of them received ABDC inhalation and their characteristics at baseline and day 1 of respiratory colonization did not differ from those of colonized patients not receiving treatment (n = 62). The ABDC-treated group had a similar *Candida* spp. load but did not decolonize more rapidly as compared to untreated patients. The clinical pulmonary infection and lung injury scores did not decrease as in the untreated group. In a Cox proportional hazard model, the duration of mechanical ventilation was increased (P < 0.003) by ABDC treatment independently of other potential determinants and *Candida* spp. colonization. No differences in ventilator-associated pneumonia or in overall mortality (up to day 90) were observed.

**Conclusion:**

Treatment of respiratory *Candida* spp. colonization in non-neutropenic critically ill patients by inhaled ABDC may not affect respiratory colonization but may increase duration of mechanical ventilation, because of direct toxicity of the drug on the lung.

**Electronic supplementary material:**

The online version of this article (doi:10.1186/s12879-014-0575-3) contains supplementary material, which is available to authorized users.

## Background

In critically ill patients, *Candida* spp. are frequently cultured from non-sterile body sites. However, the clinical significance hereof is not easy to define. When *Candida* spp. are isolated from the respiratory tract, discriminating between relatively harmless colonization or invasive infection is still surrounded by controversy and leads to therapeutic dilemmas [1]-[8]. On the one hand, respiratory isolation of *Candida* spp. could reflect the patient’s state of immunoparalysis and the elimination of normal flora through previous antibiotic treatment but may otherwise be relatively harmless. On the other hand, it could reflect a risk factor for invasive *Candida* spp. infections, even in non-neutropenic patients, such as candidemia or pulmonary candidiasis, a rare and difficult to diagnose deep infection in the critically ill [2],[3]. Respiratory *Candida* spp. colonization has also been suggested to be a risk factor for (multidrug-resistant) Gram-negative airway infection, i.e. ventilator-associated pneumonia (VAP) or tracheobronchitis, to prolong ventilator dependency and to increase mortality, even in the absence of direct pulmonary pathogenicity [6],[9]-[11].

One way to examine the clinical role of *Candida* spp. colonization of the respiratory tract is to examine the effect of treatment aimed at eradicating the fungus. The criteria to start antifungal drugs continue to be debated [1],[4]. Nevertheless, one study suggests that systemic antifungal treatment may help to prevent *Pseudomonas* spp.-associated VAP in the critically ill patient colonized by *Candida* spp. in the respiratory tract [11]. Also, in neutropenic hematology patients at risk for invasive fungal infections inhalation therapy with liposomal amphotericin B has been proven to prevent invasive pulmonary aspergillosis [12],[13]. ABDC inhalation is also used to prevent pulmonary aspergillosis in many lung transplant units but the safety and efficacy of this approach has never been validated in a large randomized placebo-controlled trial.

Selective decontamination of the digestive tract (SDD) is a strategy used in many Dutch intensive care units (ICU’s) and consists of oral administration of non-absorbable antibiotics. When *Candida* spp. respiratory colonization is documented during routine surveillance cultures of the respiratory tract and the patient is at risk for deep infection, intensivists often start with amphotericin-B deoxycholate (ABDC) inhalation therapy via a nebulizer. This treatment is started as part of many SDD protocols used in large trials [14],[15]. However, its safety and efficacy are not well established, in the absence of randomized trials [16]. In rats, aerolized ABDC decreases activity of surfactant suggesting a possibility to harm [17]-[19]. In humans, however, (liposomal) amphotericin-B inhalation may not alter surfactant but may be cytotoxic by other means [20],[21].

To further elucidate the significance of *Candida* spp. in respiratory secretions and the safety and efficacy of ABDC inhalation therapy, we retrospectively compared clinical courses of mechanically ventilated patients colonized with *Candida* spp. in the respiratory tract which received ABDC inhalation with those of a comparable group of patients in which ABDC was not initiated. The hypothesis was that ABDC is a safe and effective treatment for *Candida* spp. colonization of the respiratory tract and thereby prevents VAP and prolonged need for mechanical ventilation during SDD in the ICU.

## Methods

### Patients

**Figure 1:**
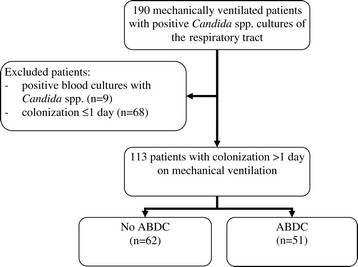
**Patient inclusion.** ABDC = amphotericin-B deoxycholate.

Data were retrospectively collected from our patient data management system, according to a predefined checklist. All patients >18 years of age requiring mechanical ventilation >48 h in the ICU of the Erasmus Medical Center Rotterdam, with at least 2 consecutive daily positive *Candida* spp. cultures of the respiratory tract (throat, tracheal aspirates or bronchoalveolar lavage), defining respiratory colonization, were included when admitted between December 2010 and December 2011. The ICU is a tertiary care mixed medical-surgical ICU with 2000 admissions per year. Patients with neutropenia (leukocyte count <0.5 × 10^9^/L), positive blood cultures for *Candida* spp., immunodeficiency (HIV, solid cancer, hematologic malignancy, solid- organ or bone marrow transplantation, or long term or high dose steroid treatment) or who did not meet respiratory colonization criteria were excluded (Figure 1). Informed consent was not needed according to the Dutch law because of the retrospective analysis in which data collected during routine clinical care were used and anonymously treated.

### Clinical protocol

Patients requiring mechanical ventilation for more than 48 h receive SDD in our unit. This involves administration of an oral paste, a suspension via the nasogastric tube and a suppository, containing the non- absorbable antibiotics tobramycin, amphotericin B and colistin. For the first three days, patients also receive cefotaxim intravenously at 4 times 1 gram a day. At the start of SDD, cultures are taken (inventory cultures) from throat, tracheal aspirates, urine, rectum and drains or wounds. To monitor the effect of the SDD, surveillance cultures (from throat, tracheal aspirates, urine, and rectum) are done routinely three times per week. SDD and surveillance cultures are continued until ICU death or discharge. On indication, additional cultures of the respiratory tract are taken at the discretion of the attending intensivist. Standard microbiological methods were used to culture the specimens. For the purpose of fungal detection in respiratory samples a Sabouraud agar (SB), incubated at 26°C was used. A Gram strain was performed to identify epithelial cells and bacteria. If the Gram strain contained 10 times more leukocytes than epithelial cells and if there were more than 6 species of one type of microorganism the streaked agar plates were incubated. *Candida* colonies are visually identified on the Sabouraud agar and identified to the species level using CHROMagar™. ABDC inhalation therapy is not part of the standard of care SDD regimen but can be added at the discretion of the attending intensivist when tracheal aspirates or throat cultures are repeatedly positive for *Candida* spp. and the patient is considered to be at risk for deep infection. For this purpose ABDC, dissolved in dextrose 5% with a final concentration of 10 mg/mL is nebulized in a Maquet nebulizing system (Maquet Servo Ultra Nebulizer, Maquet, Sweden), 4 times daily (daily dose 4 × 4 mL =40 mg). ABDC inhalation therapy is discontinued after two consecutive daily respiratory tract cultures or after detubation of the patient. Other antifungal therapy was instituted at the discretion of the attending intensivist based on cultures taken from other body sites. Pneumonia was diagnosed and treated by the attending intensivist on the basis of clinical, imaging and microbiological data in close collaboration with the infectious disease specialists. VAP was defined as the presence of a new or progressive pulmonary infiltrate on the chest radiograph along with infectious signs such as fever ≥38.5°C or ≤36.5°C, leukocyte count ≥10 × 10^9^/L or ≤4 × 10^9^/L and purulent tracheobronchial secretions [22].

### Study protocol and data collection

At admission/baseline, demographic data and clinical variables, including age, sex, pre-morbidity, prior use of antibiotics including SDD and other risk factors for *Candida* spp. colonization, antifungal treatment, steroids, immune status (active malignancy or other causes of an immunocompromised state), reasons for admission, invasive procedures (arterial or venous central catheter, pulmonary artery catheter, endotracheal intubation) and treatment of organ failure (inotropic support, hemodialysis and mechanical ventilation settings) were recorded (but not all data are shown when considered not contribute). The simplified acute physiology score (SAPS II) was computed at admission. Day 1 of the study was defined as the first day of respiratory colonization with *Candida* spp. The acute physiology and chronic health evaluation II (APACHE II) and the sequential organ failure assessment (SOFA) were recorded at day 1. All cultures from the respiratory tract were recorded from day 1 until day of decolonization, i.e. the duration of respiratory colonization. Airway *Candida* decolonization was defined as the first day of 2 consecutive daily respiratory cultures negative for *Candida* spp. in the ICU. *Candida* spp. load was calculated as the number of positive respiratory cultures and the sum of the load (in thousands of colonies) of the cultures divided by the total cultures taken, using cultures from day 1 until decolonization, ICU death or discharge, whatever came first. *Candida* spp. load was classified as mild, <10^4^, moderate, 10^4^-10^5^, and severe, >10^5^. For the ABDC-treated group, the duration of respiratory colonization and load were calculated from day 1 until start of ABDC and from the start of ABDC until decolonization, ICU death or discharge, whatever came first. In order to calculate the clinical pulmonary infection score (CPIS) [23] at day 1 and 7, routinely obtained temperature (°C), white blood cell count (WBC, 10^9^/L), amount of tracheal secretions, chest radiography imaging results and P_a_O_2_/F_I_O_2_ ratio (kPa) were recorded using the worse data recorded during the 24 hours of that day. At day 1 and 7, we also recorded positive end-expiratory pressure (PEEP, cm H_2_O), ratio of arterial PO_2_ to inspiratory O_2_ fraction (P_a_O_2_/F_I_O_2,_ kPa), total respiratory dynamic compliance (mL/cm H_2_O) and chest radiography imaging results to calculate the lung injury score (LIS) [24]. The compliance is calculated from tidal volume and difference between in- and expiratory pressures on the ventilator. The LIS is calculated by using the worst physical data recorded during the 24 hours of that day and the number of quadrants (0–4) on the chest radiograph showing alveolar consolidations. Patients were followed until day 28 and 90 after day 1 and duration of ICU stay, duration of colonization and mechanical ventilation (from day 1 until start of inhalational ABDC and thereafter until detubation not requiring reintubation within 48 h) and vital outcomes were recorded.

### Statistical analysis

Continuous variables were expressed as the mean with standard deviation (SD) or when the assumption of normality (Kolmogorov-Smirnov test P < 0.05) was violated as median values and interquartile ranges. Since ABDC treatment was started at median 5 days of colonization, patients colonized for 5 days or more were selected from the non-treated group for comparisons of durations. Kaplan-Meier curves were constructed and log rank testing was performed to evaluate group differences in colonization and ventilation durations, censored for death or discharge from the ICU. Multiple Cox proportional hazard modeling was done to evaluate the effect of ABDC treatment, irrespective of other variables associated with mechanical ventilation. Hazard ratios (HR) and 95% confidence intervals (CI) were calculated. All tests were two-sided and P < 0.05 was considered statistically significant. Exact P values >0.001 are given.

## Results

Characteristics of patients who received ABDC (n = 51) and those who did not (n = 62) are summarized in Table 1. There tended to be more males in the treated group, but groups were otherwise comparable at baseline up to day 1, suggesting similar general risks for respiratory *Candida* spp. colonization. However, ABDC-treated patients had a longer duration of stay in the ICU but mortality did not differ from that in untreated patients.

**Table 1:** **Patient characteristics at admission, day 1 of colonization and clinical course**

	ABDC no n = 62	ABDC yes n = 51	P
Age, years	57 (17)	58 (20)s	0.62
Gender, male	34 (55)	37 (73)	0.05
Premorbidity			
Cardiac	31 (50)	18 (35)	0.12
Gastointestinal	21 (34)	24 (47)	0.16
Cancer	17 (27)	16 (31)	0.65
Pulmonary	16 (26)	13 (25)	0.97
Neurological	11 (18)	12 (24)	0.45
DM	12 (19)	8 (16)	0.61
Renal	5 (8)	9 (18)	0.14
Immune	2 (3)	0 (0)	0.16
Other	20 (32)	14 (27)	0.58
Reasons of ICU admission			0.28
Suspected infection	18 (29)	18 (35)	
Respiratory failure	9 (15)	13 (25)	
Shock	9 (15)	6 (12)	
Neurological	11 (18)	4 (8)	
Medical	3 (5)	3 (6)	
Postoperative	6 (9)	4 (8)	
CPR	4 (6)	1 (2)	
Trauma	2 (3)	2 (4)	
SAPS II	43 (18)	48 (16)	0.12
**At day 1 of colonization**			
APACHE II	21 (12)	20 (9)	0.21
SOFA	9 (6)	9 (5)	0.18
Days from admission ICU	1 (3)	1 (2)	0.64
Ventilation from admission, days	1 (2)	1 (3)	0.18
**Course**			
Duration of stay ICU, days	10 (12)	24 (18)	<0.001
28-day mortality	19 (32)	10 (20)	0.13
90-day mortality	27 (45)	19 (37)	0.40

Numbers (percentage) or median (interquartile range), where appropriate. Abbreviations: ABDC = amphotericin-B deoxycholate; DM = diabetes mellitus; ICU = intensive care unit; CPR = cardiac pulmonary resuscitation; SAPS II = simplified acute physiology score; APACHE II = acute physiology and chronic health evaluation II; SOFA = sequential organ failure assessment score.

### Respiratory Candida spp. colonization, risk factors, bacterial infection and treatment characteristics according to ABDC inhalation treatment

**Figure 2:**
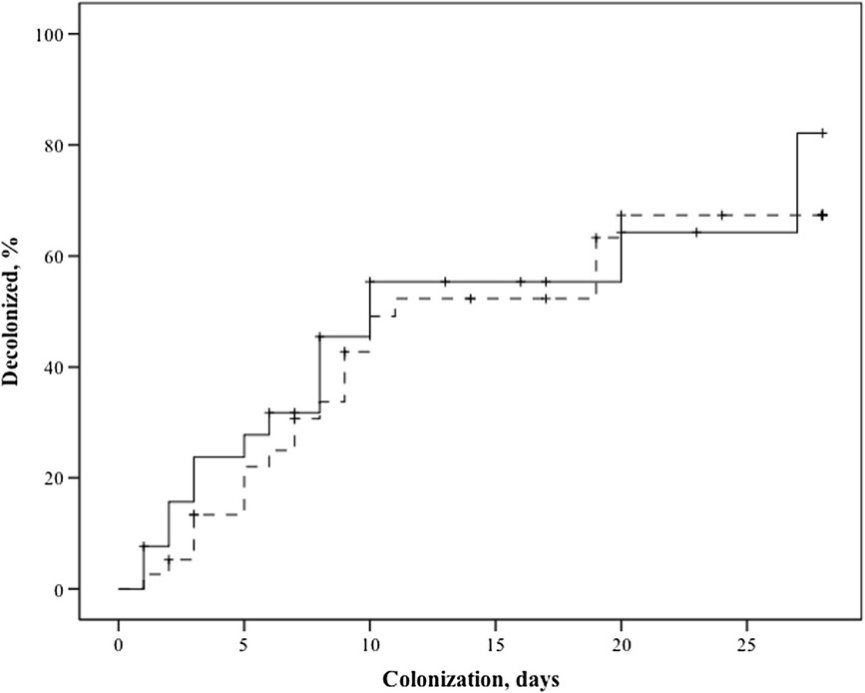
**Duration of respiratory colonization from day 5 (now day 0 in graph) of colonization in untreated (continuous line) and from start of amphotericin B (ABDC) on day 0 in graph in treated patients (dotted line) until negative cultures, censored for death or discharge from the intensive care unit, to day 28; P = 0.45 (log-rank test).**

Table 2 gives the data from day 1 of respiratory colonization until ICU death or discharge for the untreated and treated groups. Colonization lasted 5 (9) days in the untreated group and ABDC was started 5 (5) days after the day of first positive respiratory *Candida* spp. culture, so that we also compared groups for these comparable time frames. The data suggest similar risk and load of respiratory colonization with *Candida* spp. until treatment is started for both groups. However, the ABDC-treated group seemed to have an overall higher load and prolonged colonization than the untreated group, because of start of early and spontaneous decolonization in the latter. The CPIS score was higher 1 week after day 1 in the absence of more VAP in treated patients. Conversely, there was no decrease in the occurrence of VAP in the treated group. Even though total *Candida* spp. cultures in the respiratory tract after start of ABDC (1 (2)) was lower than before start (3 (1)) (P < 0.001), respiratory colonization durations were similar, from day 5 (untreated group) or start of treatment (ABDC-treated group) to 28 days later (Figure 2).

**Table 2:** **Respiratory** ***Candida*** **spp. colonization, risk factors, bacterial infection and treatment characteristics starting day 1 of colonization in untreated group and from day 1 of colonization to start of treatment in treated group and thereafter, until ICU death or discharge**

	ABDC no Day 1 until ICU death or discharge, n = 62	ABDC yes Day 1 to start treatment, n = 51	P vs no	ABDC yes Day 1 until ICU death or discharge, n = 51	P vs no
Total *Candida* spp. cultures respiratory tract 2 (1)		3 (1)	0.67	4 (3)	<0.001
*Candida* spp. load			0.46		0.36
Mild, <10^4^	38 (61)	32 (58)		30 (60)	
Moderate, 10^4^-10^5^	13 (21)	18 (33)		16 (32)	
Heavy, >10^5^	11 (18)	5 (9)		4 (8)	
*Candida albicans*	41 (66)	34 (61)	0.45	32 (63)	0.74
*Candida non-albicans*	3 (4)	1 (2)		3 (6)	
Both *Candida* spp.	18 (30)	20 (37)		16 (31)	
Duration of respiratory colonization, days	5 (9)	5 (5)	0.66	12 (18)	<0.001
Corticosteroids	23 (37)	18 (35)	0.85	22 (43)	0.52
TPN	21 (34)	16 (31)	0.78	18 (35)	0.88
Ventilator-associated pneumonia,	23 (37)	11 (22)	0.16	18 (35)	0.96
Days after first *Candida* spp. culture	4(6)	3 (4)	0.43	5(4)	0.91
Gram positive	6 (10)	0 (0)		2 (4)	
Gram negative	17 (27)	11 (22)		16 (31)	
CPIS, day 1	7 (3)	8 (2)	0.10	NA	NA
day 7	5 (4)	NA	NA	8 (3)	<0.001
Systemic antifungal treatment	17 (27)	13 (26)	0.39	18 (35)	0.93
Echinocandins	11 (17)	9 (18)		11 (21)	
Azoles	6 (10)	4 (8)		7 (14)	

Numbers (percentage) or median (interquartile range), where appropriate. Abbreviations: ABDC = amphotericin-B deoxycholate; TPN = total parenteral nutrition; CPIS = clinical pulmonary infection score; day 1 and 7 refer to day of first isolation of *Candida* spp. in respiratory secretions and 1 week later, respectively; NA = not applicable.

### Patient courses

**Figure 3:**
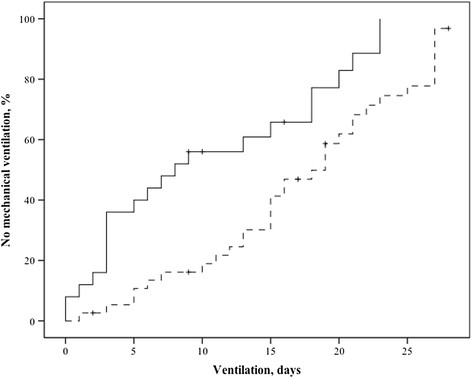
**Duration of mechanical ventilation from day 5 (now day 0 in graph) of colonization in untreated (continuous line) and from start of amphotericin B (ABDC) on day 0 in graph in treated patients (dotted line), censored for death or discharge from the intensive care unit, to day 28; P = 0.003 (log-rank test).**

Table 3 gives the ventilation characteristics for both groups. The LIS and P_a_O_2_/F_I_O_2_ ratio at day 1 were similar between the groups, but at day 7 the LIS was higher and the P_a_O_2_/F_I_O_2_ ratio was lower in the ABDC-treated than untreated group. The PEEP was higher in the ABDC-treated group at day 7. Patients receiving ABDC were mechanically ventilated longer than patients without treatment (Figure 3), from day 5 or start of treatment onwards, in untreated and treated groups, respectively. The effect of ABDC on mechanical ventilation duration was independent from other factors such as duration of colonization after day 5 and LIS on day 7 (Table 4).

**Table 3:** **Ventilation characteristics at day 1 and 7 of colonization**

	ABDC no n = 62	ABDC yes n = 51	P
Tidal volume, ml/kg, day 1	7 (2)	7 (2)	0.77
Tidal volume, ml/kg, day 7	7 (3)	8 (3)	0.50
PEEP, cm H_2_O, day 1	9 (4)	9 (4)	0.10
PEEP, cm H_2_O, day 7	6 (5)	10 (6)	0.004
Compliance, mL/cm H_2_O, day 1	37 (28)	44 (39)	0.05
Compliance, mL/cm H_2_O, day 7	40 (27)	41 (36)	0.09
P_a_O_2_/ F_I_O_2_ ratio, day 1	213 (122)	197 (137)	0.40
P_a_O_2_/ F_I_O_2_ ratio, day 7	282 (123)	217 (147)	0.005
Chest X-ray, no. of quadrants, day 1	2 (2)	2 (1)	0.27
Chest X-ray, no. of quadrants, day 7	2 (3)	2 (2)	0.41
LIS, day 1	2.0 (1.0)	1.8 (1.3)	0.47
LIS, day 7	1.0 (1.3)	1.8 (1.5)	0.002
Ventilation after day 1, days	7 (9)	20 (16)	<0.001

Numbers (percentage) or median (interquartile range), where appropriate. Abbreviations: ABDC = amphotericin B deoxycholate; PEEP = positive end-expiratory pressure; P_a_O_2_ = partial pressure of oxygen in the blood (kPa); F_I_O_2_ = the percentage of oxygen administered; LIS = lung injury score; day 1 and 7 refer to day of first isolation of *Candida* spp. in respiratory secretions and 1 week later, respectively.

**Table 4:** **Multiple Cox proportional hazard modeling for duration of mechanical ventilation**

	HR (95% CI)	P
ABDC treatment	3.63 (1.57-8.34)	0.003
*Candida* spp. load until day 5	1.19 (0.70-2.02)	0.51
*Candida* spp. load after day 5	0.67 (0.31-1.47)	0.32
Duration of colonization until day 5	0.96 (0.90-1.02)	0.13
Duration of colonization after day 5	0.92 (0.88-0.96)	<0.001
CPIS day 7	0.91 (0.76-1.10)	0.32
LIS day 7	0.60 (0.40-0.91)	0.02

Hazard ratios (HR; 95% confidence interval, CI) are given for each variable. Abbreviations: ABDC = amphotericin B deoxycholate; CPIS = clinical pulmonary infection score; LIS = lung injury score.

## Discussion

This non-randomized observational study suggests that ABDC inhalation by critically ill, mechanically ventilated patients with respiratory *Candida* spp. colonization is not effective and potentially harmful. Patients on ABDC inhalation did not decolonize more rapidly, remained mechanically ventilated for an additional 13 days and had a longer ICU stay but no increased mortality, as compared to untreated patients.

Our study suffers from a non-randomised design, so that conclusions should be drawn carefully. Nevertheless, the data suggest that prior to start of treatment, disease severity, colonization risk and load were similar among the groups. Treatment was started approximately 5 days after the first positive respiratory *Candida* spp. culture, when in the untreated group spontaneous decolonization had already started. We are therefore uncertain, in disagreement with previous studies [16], whether decolonization would have also occurred in the treated group if treatment had been disadvised or postponed. However, when groups were matched for the same duration of respiratory colonization prior to treatment, no difference could be observed in rate of decolonization (Figure 2), in contrast to the increase in decolonization with treatment from 62 to 86% observed in a previous, but larger, non-randomized study [16].

A previous study showed an incidence of VAP in patients with respiratory *Candida* spp. colonization of 24% which is comparable to the 29% incidence in this study [9]. However, our data do not suggest that *Candida* spp. colonization in the respiratory tract predisposed to VAP, in contrast to studies suggesting that this could promote VAP development, especially when caused by *Pseudomonas aeruginosa* and multidrug resistant bacteria [9],[10]. Conversely, there is no suggestion that respiratory decolonization was associated with less VAP, in contrast to suggestions that systemic antifungal treatment decreases the incidence of VAP [11]. The differences in ventilation durations cannot be attributed to differences in the occurrence of VAP, even though the relatively non-specific CPIS (as well as the LIS) was higher on day 7 in the ABDC-treated group, since the CPIS was not a determinant of mechanical ventilation duration in multivariate analysis.

Safety issues of ABDC inhalation therapy remain a concern [19],[20]. We therefore looked at pulmonary injury and ventilation durations, which appeared independent of respiratory colonization duration in our patients. The lung scores suggest greater injury in treated patients at day 7 already than in untreated patients, independent of VAP. Two studies showed surfactant dysfunction by ABDC, thereby deteriorating gas exchange [17],[18], possibly via direct inhibition or damage of the alveolar capillary membrane resulting in an influx of surfactant-inactivating plasma proteins. Loss of surfactant could lead to longer requirement of mechanical ventilation in patients receiving inhalational ABDC. Indeed, the longer duration of mechanical ventilation and thus ICU stay in our study in ABDC-treated patients can be explained by pulmonary adverse effects of the drug. Our data do not exclude that ABDC inhalation prevents pulmonary infections with *Aspergillus* spp. but suggests that it may carry pulmonary toxicity.

## Conclusions

In conclusion, treatment of pulmonary Candida spp. colonization in non-neutropenic critically ill patients by inhaled ABDC may not facilitate respiratory decolonization but may increase duration of mechanical ventilation, because of direct pulmonary toxicity of the drug. Therefore, inhalation of ABDC for respiratory colonization with *Candida* spp. in non-neutropenic critically ill patients cannot be recommended outside prospective randomized trials. For the latter, the use of less toxic liposomal dissolved formulation may be preferred [21],[25].

### Ethics

This study does not need approval from a medical ethical committee for the following reason. Division 1, Section 1.1.b. of the Dutch law on medical research defines medical research as research in which persons are subjected to treatment or are required to follow a certain behavioral strategy (www.ccmo.nl, http://www.ccmo.nl/attachments/files/wmo-engelse-vertaling-29-7-2013-afkomstig-van-vws.pdf). This means that retrospective patient data analysis is not subject to this law. Indeed, informed consent is not needed, provided that, as we did, data are retrieved and analyzed anonymously.

## Authors’ contributions

PG, ED and AG participated in the design of the study. PG, ED and BR extracted data from the information systems and made the case record forms. Statistical analysis was performed by PG and AG. All authors read and approved the final manuscript.

## Authors’ original submitted files for images

Below are the links to the authors’ original submitted files for images. Authors’ original file for figure 1 Authors’ original file for figure 2 Authors’ original file for figure 3
