# Supplementary figures and images for: Safety and efficacy of amphotericin-B deoxycholate inhalation in critically ill patients with respiratory Candida spp. colonization: a retrospective analysis
Source: BMC Infect Dis. 2014 Oct 28;14:575. doi: 10.1186/s12879-014-0575-3 (PMC4213474; doi:10.1186/s12879-014-0575-3)

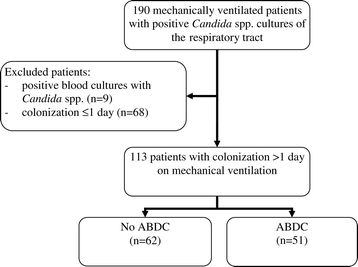

Supplement: Supplementary file 1 — Authors’ original file for figure 1 [file 12879_2014_575_MOESM1_ESM.gif]

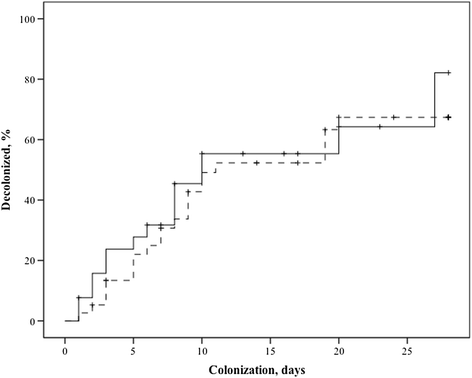

Supplement: Supplementary file 2 — Authors’ original file for figure 2 [file 12879_2014_575_MOESM2_ESM.gif]

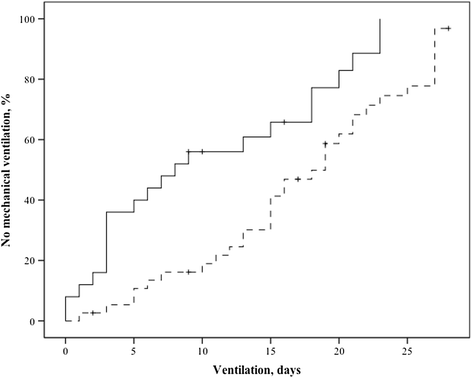

Supplement: Supplementary file 3 — Authors’ original file for figure 3 [file 12879_2014_575_MOESM3_ESM.gif]
